# Viral Respiratory Infections and Host Immune Dynamics in Diabetes: Clinical Outcomes in the Post-COVID Era

**DOI:** 10.3390/microorganisms14071476

**Published:** 2026-07-06

**Authors:** Ana Maria Mihai, Florina Cristiana Lucaciu, Ovidiu Rosca, Daniel Alexandru Jipa, Monica Cialma, Andra-Elena Saizu, Andreea Cristina Floruncut, Andrada Tarau, Alexandra Sima

**Affiliations:** 1 Doctoral School, “Victor Babes” University of Medicine and Pharmacy Timisoara, Eftimie Murgu Square 2, 300041 Timisoara, Romania; ana-maria.mihai@umft.ro (A.M.M.);; 2 Department XIII, Discipline of Infectious Diseases, “Victor Babes” University of Medicine and Pharmacy Timisoara, Eftimie Murgu Square 2, 300041 Timisoara, Romania; 3 Victor Babes Clinical Hospital for Infectious Diseases and Pneumology of Timisoara, 300041 Timisoara, Romania; 4 Department of Diabetes, Nutrition and Metabolic Diseases Clinic, “Pius Brînzeu” Emergency Clinical County University Hospital, 300723 Timisoara, Romania

**Keywords:** viral co-infection, diabetes mellitus, interleukin-6, post-COVID era, multiplex PCR, neutrophil-to-lymphocyte ratio, procalcitonin

## Abstract

The introduction of respiratory multiplex PCR in the post-pandemic world has improved the detection of viral infections, whose clinical relevance is still being characterized. Patients with diabetes mellitus (DM) exhibit altered innate immune responses, yet the effect of concurrent viral infection on their inflammatory trajectory and clinical outcomes remains poorly characterized. This study examined whether diabetes is associated with a more pronounced inflammatory response, delayed resolution, and worse multi-organ outcomes during viral respiratory infections. A prospective, longitudinal cohort of 430 hospitalized adults (DM: n = 211; non-DM: n = 219) with PCR-confirmed viral respiratory infections was stratified into four groups by diabetes and co-infection status using a respiratory multiplex PCR panel. Serum IL-6, CRP, NLR, procalcitonin, and urea were measured at admission (Day 1) and at clinical stabilization (Day 6). All variables failed normality testing (Shapiro–Wilk *p* < 0.0001); non-parametric methods were applied. Receiver operating characteristic (ROC) analysis was used to identify candidate biomarker cutoffs for mortality prediction. SARS-CoV-2 was the predominant pathogen (29.1%). In an exploratory comparison limited by small subgroup sizes (n = 8 vs. n = 14), co-infected diabetic patients had higher baseline inflammatory markers than co-infected non-diabetic patients: median IL-6 32.87 vs. 6.20 pg/mL (Mann–Whitney *p* = 0.0006) and median CRP 103.83 vs. 23.03 mg/L (*p* = 0.0012). At the Day 6 checkpoint, co-infected diabetic survivors had higher IL-6 (12.01 vs. 6.13 pg/mL, *p* = 0.0183) and showed little within-group NLR change (Wilcoxon *p* = 0.2367); these Day 6 estimates are subject to survivor selection and should be interpreted accordingly. In-hospital mortality was 25.6% in diabetic vs. 3.7% in non-diabetic patients (*p* < 0.0001). Diabetic patients more frequently required orotracheal intubation (6.4% vs. 1.0%, *p* = 0.0207) and high-flow nasal oxygen (HFNO) support (7.9% vs. 1.8%, *p* = 0.0166). In an internal ROC analysis, baseline IL-6 showed the highest discriminatory performance for in-hospital mortality (AUC 0.812, 95% CI 0.772–0.848), with a candidate cutoff of > 55.78 pg/mL (sensitivity 71.0%, specificity 79.1%); IL-6 outperformed CRP (AUC 0.706, DeLong *p* = 0.0029) and NLR (AUC 0.656, DeLong *p* = 0.0001). As this cutoff was derived and evaluated in the same cohort, it is reported as exploratory and requires external validation. In this single-center cohort, diabetes was associated with a more pronounced baseline inflammatory profile, slower resolution of the neutrophil-to-lymphocyte ratio, and greater multi-organ involvement during viral respiratory infection, including in the small co-infected subgroup. In the full cohort, diabetes remained associated with higher mortality, IL-6, and CRP after adjustment for age, sex, and BMI; however, the small co-infected subgroups could not be adjusted, so those specific comparisons should be regarded as hypothesis-generating and need confirmation in larger, adequately powered multi-center cohorts.

## 1. Introduction

The post-pandemic respiratory landscape is characterized by the concurrent circulation of SARS-CoV-2 and re-emerging seasonal pathogens, including Influenza A/B, Respiratory Syncytial Virus (RSV), and Human Metapneumovirus, in a population whose baseline immunity has shifted [1]. The clinical use of respiratory multiplex PCR panels has reclassified findings once regarded as incidental into a recognized clinical entity: viral–viral co-infections, defined as the simultaneous detection of two or more distinct respiratory pathogens in a single patient sample [2,3,4,5]. While multiplex PCR served as our reference method, rapid antigen tests provide a faster, lower cost, point-of-care complement that can support early isolation and treatment decisions in the respiratory-virus setting, albeit at a lower sensitivity than a molecular panel [6,7]. Their role alongside multiplex platforms in stratifying diabetic patients warrants further evaluation.

Among metabolic comorbidities predisposing individuals to severe respiratory outcomes, diabetes mellitus (DM) remains foremost. Chronic hyperglycemia induces multiple immune defects: impaired phagocytic activity, disrupted neutrophil chemotaxis, attenuated interferon signaling, and a baseline state of low-grade vascular inflammation driven by advanced glycation end-products (AGEs) and sustained NF-κB activation [8,9,10,11,12]. While prior literature has extensively characterized single-pathogen responses in diabetic cohorts, particularly SARS-CoV-2 [13], the host response to a simultaneous multi-viral challenge in this metabolic context remains largely uncharacterized.

This gap can be framed as a two-hit model: a host with pre-existing, metabolically driven immune dysfunction must simultaneously mount antiviral responses against two distinct pathogens. The resulting immune response, whether additive, synergistic, or antagonistic, may have consequences for the cytokine trajectory, the capacity for inflammatory resolution, and subsequent organ injury [14,15].

This prospective longitudinal study addresses three sequential hypotheses: First, that a diabetic background is associated with a more pronounced inflammatory response at admission when two viral pathogens are present concurrently, compared with non-diabetic counterparts. Second, that the resolution rate of inflammatory markers by Day 6 is impaired in co-infected patients with diabetes, who remain with elevated IL-6. Third, that this inflammatory profile is associated with greater clinical severity, assessed by the incidence of respiratory failure, renal injury, procalcitonin elevation as a marker of secondary bacterial infection risk and antimicrobial use [15,16]. As a secondary, exploratory aim, we performed ROC analysis to examine whether baseline inflammatory markers, and IL-6 in particular, could be used to derive a candidate threshold for in-hospital mortality, with the understanding that any such cutoff would require independent validation before clinical use.

## 2. Materials and Methods

### 2.1. Study Design and Patient Cohort Allocation

This prospective, longitudinal investigation was conducted in a single medical hospital in western Romania (December 2024–April 2026). A cohort of 430 adult patients requiring formal inpatient admission for acute, clinically significant viral respiratory infections was enrolled. Inclusion criteria mandated respiratory symptom presentation (fever ≥ 38 °C, dyspnea, productive cough, or hypoxemia with SpO_2_ < 95%) with etiology confirmed by multiplex PCR within 12 h of admission [17]. Exclusion criteria were age under 18, failure to confirm the infection by multiplex PCR and absence of informed consent. The study protocol was approved by the institutional Ethics Committee and internal review board (Approval No. 12066/12 December 2024). All participants provided written informed consent prior to enrollment.

Patients were stratified into four analytical groups based on their diabetes mellitus history and co-infection status: Group 1: Non-Diabetic Mono-infected (n = 205); Group 2: Non-Diabetic Co-infected (n = 14); Group 3: Diabetic Mono-infected (n = 203); and Group 4: Diabetic Co-infected (n = 8). The overall co-infection rate was 5.1% (22/430), consistent with post-pandemic multiplex PCR surveillance data from comparable European centers [2]. The distribution of cases across the study period was: 2024 (14.2%, n = 61), 2025 (67.7%, n = 291), and 2026 (18.1%, n = 78). All eligible consecutive admissions during the study period were enrolled; no matching, propensity selection, or quota-based recruitment was applied. The near-identical size of the non-diabetic and diabetic mono-infection groups (n = 205 and n = 203) is therefore coincidental and reflects the natural distribution of diabetes among adults hospitalized with viral respiratory infection at our center during the enrolment window.

### 2.2. Virological Detection and Multiplex PCR

Molecular testing was performed using the BioFire FilmArray Respiratory Panel 2.1, a closed, automated multiplex nested PCR system that simultaneously detects the following targets: Influenza A/H3N2, Influenza A/H1N1-2009, Influenza A (untyped), Influenza B, Respiratory Syncytial Virus, Rhinovirus/Enterovirus, SARS-CoV-2, Adenovirus, Human Coronaviruses OC43, NL63, 229E and HKU1, Human Metapneumovirus, and Parainfluenza viruses 1–4 [18,19]. Sample preparation, amplification and target detection were performed strictly according to the manufacturer’s instructions for use [17]. Detection of each target is reported by the FilmArray software as a binary call (detected/not detected), based on the proprietary post-amplification melt-curve analysis and internal quality-control criteria. A viral–viral co-infection was therefore defined as a single nasopharyngeal sample with two or more distinct respiratory viral targets simultaneously reported as detected by the assay. Samples flagged as invalid by the internal controls were repeated.

### 2.3. Host Immune Biomarkers and Clinical Outcome Parameters

Venous blood was collected at Day 1 (within 12 h of admission) and Day 6 (clinical stabilization checkpoint). Interleukin-6 (IL-6, pg/mL) and high-sensitivity C-reactive protein (CRP, mg/L) were measured at both timepoints. The neutrophil-to-lymphocyte ratio (NLR) was derived from automated differential leukocyte counts. Procalcitonin (PCT, ng/mL) and serum urea (mg/dL) were measured as secondary outcome markers for bacterial superinfection risk and renal function, respectively. The number of concurrent antibiotic prescriptions and total length of stay were recorded as resource-use outcomes. Organ-system complications were recorded as binary outcomes: respiratory failure, cardiovascular failure, renal/urinary failure, and in-hospital mortality (defined as discharge status of “deceased”). Respiratory support was categorized as nasal oxygen, CPAP, high-flow nasal oxygen (HFNO) or orotracheal intubation with mechanical ventilation.

Antibiotic initiation was not protocol-mandated by study design; in clinical practice it was guided by a combination of treating-physician clinical suspicion of bacterial co-infection, procalcitonin values, and microbiological results (blood/sputum cultures, in line with institutional stewardship practice). Antibiotic counts are reported as an observational resource-use outcome and do not imply microbiologically confirmed bacterial infection in every case.

### 2.4. Statistical Analysis

All statistical analyses were performed using MedCalc Statistical Software (version 23.5.5, MedCalc Software Ltd., Ostend, Belgium). Distribution normality was assessed using the Shapiro–Wilk test; all continuous biomarkers demonstrated severe right-skewed non-normal distributions (W range 0.13–0.82, *p* < 0.0001 for all), confirming the requirement for non-parametric methods. Results are expressed as median (IQR, 25th–75th percentile) for continuous and n (%) for categorical variables. Global comparisons across the four groups used the Kruskal–Wallis H test with Conover post-hoc analysis; pairwise comparisons between Groups 4 and 2 used the Mann–Whitney U test (two-sided, exact method). Categorical outcomes were compared using chi-square testing, with Fisher’s exact test applied when expected cell counts were below 5. Longitudinal within-patient changes from Day 1 to Day 6 were assessed by the Wilcoxon signed-rank test. ROC curve analysis identified optimal mortality-prediction cutoffs (Youden index method); AUC values were compared using the DeLong method. A two-sided alpha of *p* < 0.05 was defined as the significance threshold.

To assess whether associations between diabetes and outcomes were independent of age and other baseline factors, we performed multivariable analyses on the full cohort (n = 430). In-hospital mortality was modeled by multivariable logistic regression (covariates: diabetes, age, sex, body mass index); baseline IL-6 and CRP, which were non-normally distributed, were modeled by median (quantile) regression with the same covariates. Odds ratios (ORs) and adjusted median differences are reported with 95% confidence intervals.

## 3. Results

### 3.1. Baseline Demographic and Epidemiological Characteristics

**Figure 1:**
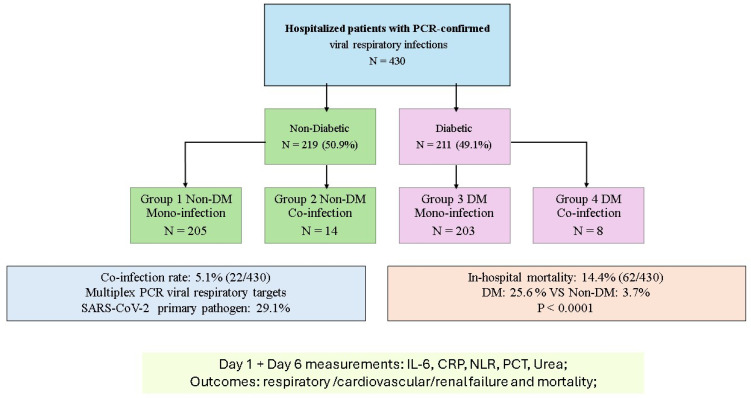
Study population flowchart.

The flow of patients through the study is shown in Figure 1. Baseline demographic and clinical characteristics are summarized in Table 1. The cohort demonstrated a distinct age separation across the four groups: Group 4 patients were significantly older (median 81.0 years, IQR 78.0–84.5) compared to Group 2 (56.5 years, IQR 37.0–75.0), consistent with the known age-related prevalence of diabetes (Kruskal–Wallis *p* < 0.0001). Glycemic control was most severely impaired in Group 4, with a median HbA1c of 9.2% (IQR 7.8–10.5%), significantly exceeding the 7.2% observed in group 3 (*p* < 0.0001). Median admission glycemia followed the same gradient (10.8 vs. 5.4 mmol/L in Groups 4 vs. 2, *p* < 0.0001).

Sex distribution (*p* = 0.5219), COVID-19 vaccination coverage (*p* = 0.2904), and seasonal influenza vaccination (*p* = 0.5213) did not differ significantly across groups, making major vaccine-related confounding unlikely. Age, however, differed substantially across groups and is considered as a potential confounder in the Section 4.

### 3.2. Baseline Host Inflammatory Response

**Figure 2:**
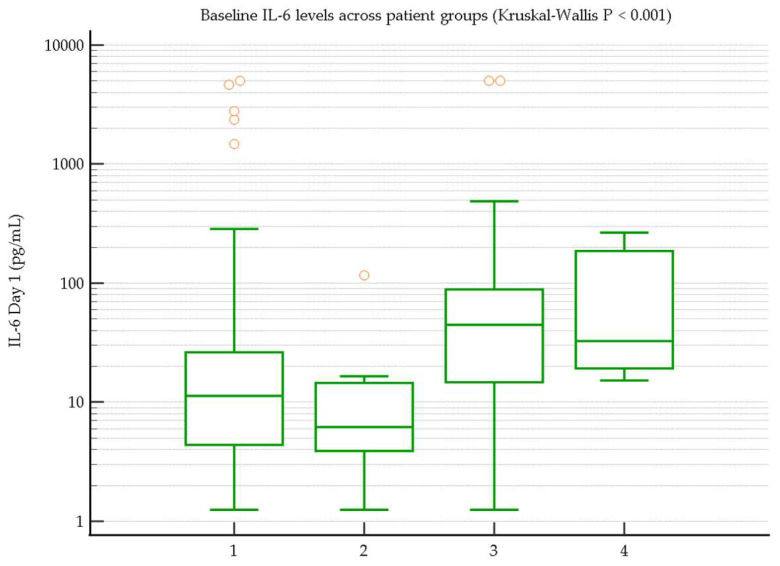
Baseline serum interleukin-6 (IL-6) levels across the four host–pathogen cohorts. Boxplots use a logarithmic Y-axis owing to extreme right-skewed distributions. The Group 4 (DM Co-infected) vs. Group 2 (Non-DM Co-infected) comparison confirms the double-hit hypothesis (Mann–Whitney U, *p* = 0.0006). Kruskal–Wallis global *p* < 0.0001.

**Figure 3:**
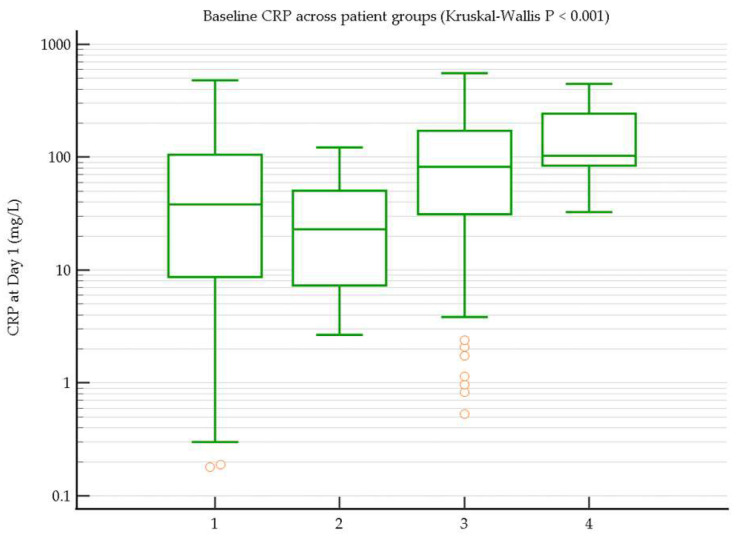
Baseline C-reactive protein (CRP) values at admission across clinical cohorts. The diabetic co-infected cohort (Group 4) exhibits a 4.5-fold median elevation to 103.83 mg/L compared to Group 2, mirroring the IL-6 amplification and confirming convergent activation of both acute-phase and cytokine pathways (Mann–Whitney U, *p* = 0.0012).

Table 2 and Figure 2 and Figure 3 present the inflammatory biomarker dynamics across all four groups. Baseline IL-6 differed significantly across groups (Kruskal–Wallis *p* < 0.0001). In an exploratory pairwise comparison between Group 4 (n = 8) and Group 2 (n = 14), co-infected diabetic patients had a 5.3-fold higher median IL-6 at admission (32.87 pg/mL, IQR 19.28–192.65) than co-infected non-diabetic patients (6.20 pg/mL, IQR 3.90–14.55; Mann–Whitney U exact *p* = 0.0006). Baseline CRP showed a 4.5-fold difference: 103.83 mg/L (IQR 84.45–246.90) in Group 4 versus 23.03 mg/L (IQR 7.32–50.51) in Group 2 (*p* = 0.0012). Baseline NLR was higher in Group 4 (5.36 vs. 3.81; *p* = 0.0698) without reaching statistical significance; given the small size of Group 4 (n = 8), this comparison should be regarded as exploratory.

The co-infection in isolation, without the diabetic metabolic background, did not significantly amplify the inflammatory response compared to non-diabetic mono-infection (Group 2 vs. Group 1 baseline IL-6: 6.20 vs. 11.32 pg/mL). This pattern is consistent with the amplified response being driven primarily by the diabetic background rather than by dual viral burden alone, although the small co-infected subgroups and the age difference between Group 2 (median 56.5 y) and Group 4 (median 81 y) preclude a firm causal interpretation.

### 3.3. Inflammatory Resolution Kinetics: Day 1 to Day 6

**Figure 4:**
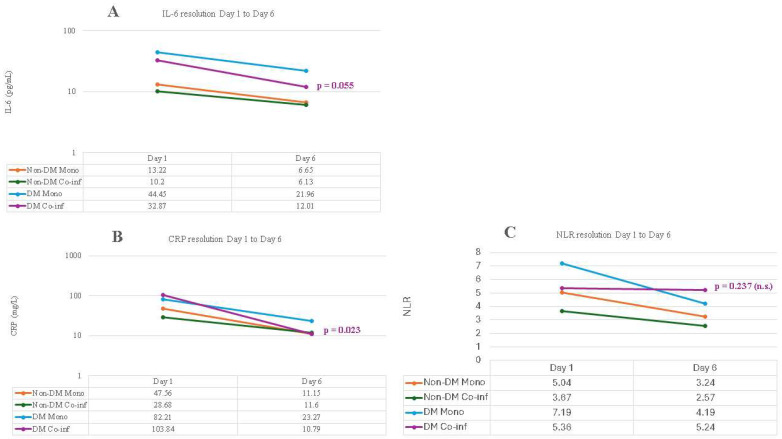
Inflammatory resolution kinetics from Day 1 to Day 6 across the four cohorts. (**A**) IL-6 trajectory (pg/mL, log scale). (**B**) CRP trajectory (mg/L, log scale). (**C**) NLR trajectory (linear scale). Each line represents the median value per group at each timepoint. The dark red Group 4 (DM Co-infected) line in Panel C remains nearly horizontal (5.36 → 5.24, Wilcoxon *p* = 0.237, n.s.), demonstrating absent NLR resolution and persistent immune exhaustion specific to this group. All other groups show significant NLR resolution (Wilcoxon *p* < 0.001).

Within-group longitudinal analysis using the Wilcoxon signed-rank test showed divergent resolution trajectories (Figure 4). All three biomarkers (IL-6, CRP, NLR) expressed significant resolution from Day 1 to Day 6 in Groups 1, 2, and 3 (all Wilcoxon *p* < 0.05 except Group 2 NLR *p* = 0.8658, attributable to the small subgroup size). The exception was Group 4: IL-6 (*p* = 0.0547) and CRP (*p* = 0.0234) trended toward resolution, and the NLR remained essentially unchanged (Day 1: 5.36 → Day 6: 5.24; Wilcoxon *p* = 0.2367, n.s.). This near-zero NLR decline in the diabetic co-infected group, contrasting with the substantial resolution observed in all other cohorts is compatible with persistent neutrophilic activation and relative lymphopenia, a pattern that has been described in the context of cellular immune dysregulation in hyperglycemic states, although our small subgroup precludes confirmation of this mechanism.

The Day 6 cross-sectional NLR comparison (Group 4 vs. Group 2: 5.24 vs. 2.57) did not reach significance (Mann–Whitney *p* = 0.2766) most likely due to the small subgroup sizes, but the absolute difference is clinically relevant. The partial resolution of CRP and IL-6 in Group 4, alongside a persistently elevated NLR, suggests that the acute-phase response resolved while the neutrophil-predominant cellular response did not. This pattern is consistent with the immune dysregulation in co-infected patients with diabetes residing predominantly at the level of cellular immunity rather than acute-phase reactants, although the small subgroup size limits firm conclusions.

### 3.4. Multi-Systemic Outcomes, Mortality, and Respiratory Support Escalation

**Figure 5:**
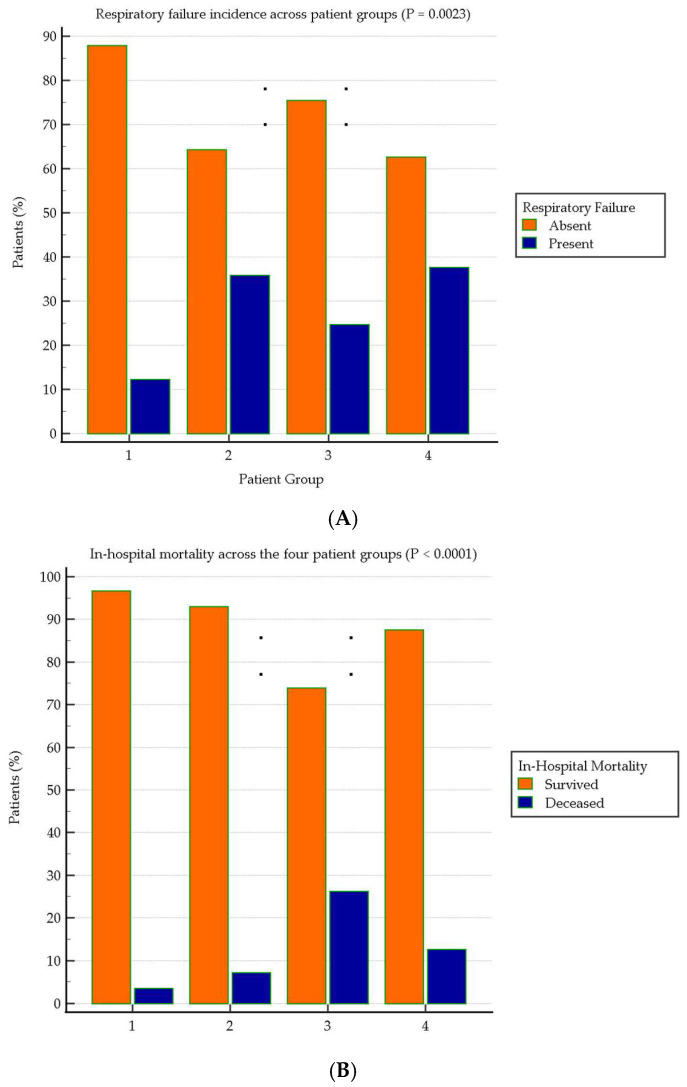
Clinical outcomes across the four patient groups. (**A**) Incidence of respiratory failure showing stepwise escalation with combined risk factors (chi-square *p* = 0.0023). (**B**) In-hospital mortality demonstrating strong diabetes-dependent gradient (chi-square *p* < 0.0001). The diabetic-dominant mortality signal is independent of co-infection status.

Secondary outcome data are summarized in Table 3 and Figure 5. The incidence of acute respiratory failure followed a stepwise escalation: 12.2% in Group 1 → 35.7% in Group 2 → 24.6% in Group 3 → 37.5% in Group 4 (chi-square *p* = 0.0023). In-hospital mortality was diabetes-dependent: 25.6% in diabetic versus 3.7% in non-diabetic patients (*p* < 0.0001), representing a seven-fold mortality increase; this difference cannot be attributed to diabetes alone and should be interpreted as descriptive. The highest mortality was observed in Group 3 (DM Mono, 26.1%) rather than Group 4 (12.5%); the apparent paradox is attributable to the small Group 4 sample (n = 8), in which complete Day 6 follow-up was achieved by survival selection. The mortality signal in this cohort was associated with diabetes status rather than with co-infection per se; given the small Group 4, no inference about an interaction between diabetes and co-infection on mortality is possible from these data.

The requirement for respiratory support followed the same diabetes-related gradient. HFNO was required in 7.9% of diabetic versus 1.8% of non-diabetic patients (*p* = 0.0166). CPAP was required in 6.2% of diabetic versus 0.9% of non-diabetic patients (*p* = 0.0080). Orotracheal intubation (mechanical ventilation) was necessary in 6.4% of Group 3 versus 1.0% of Group 1 (DM vs. non-DM overall: 6.2% vs. 0.9%, *p* = 0.0207). Notably, no co-infected patient in either Group 2 or Group 4 required mechanical ventilation, suggesting that the co-infected subgroups, although smaller, were not characterized by terminal respiratory failure requiring intubation in this cohort.

Renal strain followed the same gradient: Group 4 presented with a median urea of 82.39 mg/dL (IQR 54.57–110.21) at admission, compared to 28.89 mg/dL in Group 2 (*p* = 0.0026), reflecting acute kidney stress superimposed on pre-existing diabetic nephropathy. Procalcitonin levels in Group 4 (median 1.95 ng/mL, IQR 0.21–7.41) were 49-fold higher than in Group 2 (0.04 ng/mL; *p* = 0.0029), indicating high susceptibility to secondary bacterial translocation. This vulnerability directly drove the antimicrobial prescription burden: Group 4 required a median of 2.50 concurrent antibiotic courses (IQR 2.0–4.0) versus 1.00 in Group 2 (*p* = 0.0036).

In multivariable analysis, diabetes remained independently associated with in-hospital mortality after adjustment for age, sex, and body mass index (adjusted OR 6.98, 95% CI 3.15–15.48, *p* < 0.001), compared with an unadjusted OR of 9.07; older age was also independently associated with mortality (OR ≈ 1.06 per year, *p* < 0.001), and adding co-infection status did not change the diabetes estimate (OR 6.98). In median regression, diabetes was associated with higher baseline IL-6 (adjusted median difference +30.6 pg/mL, 95% CI 24.1–37.0, *p* < 0.001) and CRP (+46.7 mg/L, 95% CI 29.1–64.3, *p* < 0.001), independent of age, sex, and body mass index. In a sensitivity analysis restricted to patients aged 65–80 years (n = 200), in whom median age was comparable between groups (74.5 vs. 72.5 years), diabetic patients retained higher IL-6 (56.74 vs. 13.88 pg/mL, *p* < 0.001), CRP (90.44 vs. 47.88 mg/L, *p* = 0.003), and mortality (21.7% vs. 7.5%, *p* = 0.010). These analyses indicate that the diabetes-associated inflammatory and prognostic burden is not attributable to age alone.

The individual viral co-infection pairs observed among the 22 co-infected patients, together with their baseline inflammatory markers, organ involvement, and outcomes, are detailed later in Table 5.

### 3.5. ROC Analysis: IL-6 as the Optimal Mortality Predictor

**Figure 6:**
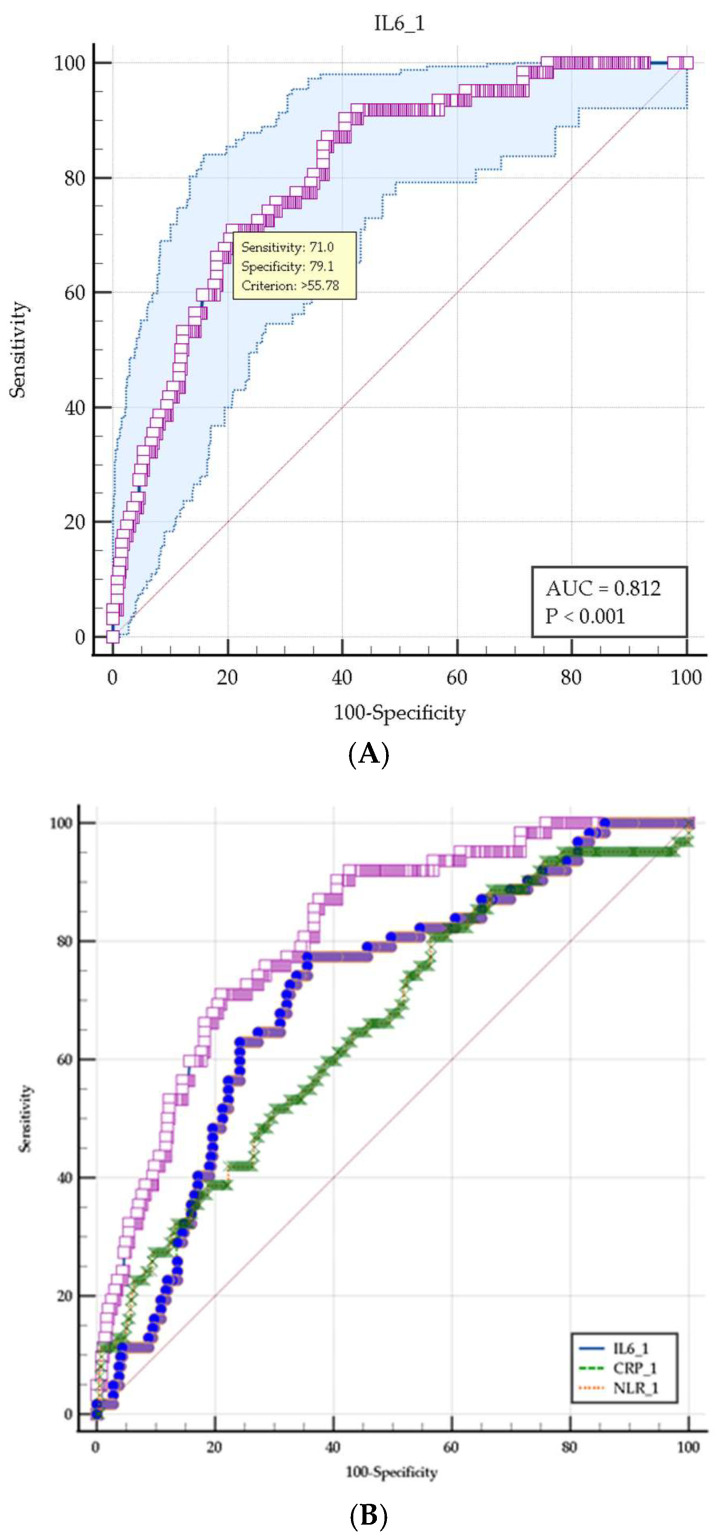
Receiver operating characteristic (ROC) curve analysis for predicting in-hospital mortality. (**A**) IL-6 at Day 1 demonstrates strong discriminatory power (AUC = 0.812, 95% CI 0.772–0.848, *p* < 0.0001). The optimal cutoff identified by Youden’s index is IL-6 > 55.78 pg/mL (sensitivity 71.0%, specificity 79.1%). (**B**) Comparison of three baseline inflammatory biomarkers as mortality predictors. IL-6 (AUC 0.812) significantly outperforms both CRP (AUC 0.706; DeLong *p* = 0.0029) and NLR (AUC 0.656; DeLong *p* = 0.0001).

To explore whether the biomarker findings could inform risk stratification, we performed ROC curve analysis for the three baseline inflammatory markers as predictors of in-hospital mortality (n = 430; 62 deaths, 14.4%). Results are presented in Table 4 and Figure 6. Baseline IL-6 showed the highest discriminatory performance among the three markers (AUC = 0.812, 95% CI 0.772–0.848, *p* < 0.0001). The optimal mortality-prediction cutoff identified by the Youden index was IL-6 > 55.78 pg/mL, providing 71.0% sensitivity and 79.1% specificity. Baseline CRP (AUC 0.706, 95% CI 0.660–0.748; optimal cutoff > 81.1 mg/L) and NLR (AUC 0.656, 95% CI 0.609–0.701; optimal cutoff > 4.22) demonstrated lower but still significant discriminatory performance.

Pairwise comparison of AUC values using the DeLong method confirmed that IL-6 significantly outperformed both alternatives: IL-6 vs. CRP (AUC difference 0.107, 95% CI 0.036–0.177, *p* = 0.0029); and IL-6 vs. NLR (AUC difference 0.157, 95% CI 0.079–0.234, *p* = 0.0001). CRP and NLR were statistically equivalent as mortality predictors (DeLong *p* = 0.1920). In this cohort, a baseline IL-6 > 55.78 pg/mL identified 71% of patients who died in hospital, with acceptable specificity, and may be useful for early risk stratification at admission. Given the single-center design and the absence of external validation, this threshold should be regarded as a candidate cutoff rather than a definitive one, and requires confirmation before use in triage algorithms.

Our candidate threshold (IL-6 > 55.78 pg/mL) sits within the mid-to-upper range of values reported for viral respiratory infection. In COVID-19 ICU cohorts, mortality-associated IL-6 cutoffs have ranged from approximately 27 to 75 pg/mL, with lower values (≈11–20 pg/mL) reported in some settings. Our somewhat higher threshold is consistent with a cohort enriched for diabetic and co-infected patients, in whom baseline IL-6 is elevated. Importantly, absolute IL-6 cutoffs are not directly transferable between studies: reported optimal thresholds vary several-fold depending on the immunoassay platform and case-mix. This reinforces that our value is a candidate cutoff requiring external, assay-standardized validation rather than a universally applicable number [20,21,22].

**Table 5:** Viral co-infection patterns and admission characteristics of the 22 co-infected patients, ordered by diabetes status and descending baseline IL-6.

Case	Diabetes Status	Age (y)	Viral Co-Infection Pair	IL-6 Day 1 (pg/mL)	CRP Day 1 (mg/L)	NLR Day 1	Organ Failure	Outcome
1	Non-diabetic	73	RSV + SARS-CoV-2	116.60	122.32	4.26	Respiratory	Died
2	Non-diabetic	37	Influenza A + Parainfluenza	16.55	31.65	2.92	—	Survived
3	Non-diabetic	36	Coronavirus NL63 + Rhinovirus	14.55	105.75	3.67	Respiratory	Survived
4	Non-diabetic	68	RSV + Rhinovirus	14.55	69.71	5.76	—	Survived
5	Non-diabetic	76	RSV + Rhinovirus	11.64	14.00	2.02	Respiratory	Survived
6	Non-diabetic	86	RSV + SARS-CoV-2	10.20	23.67	2.22	—	Survived
7	Non-diabetic	37	Parainfluenza + Rhinovirus	6.50	7.32	3.96	—	Survived
8	Non-diabetic	27	Influenza A + RSV	5.90	50.51	2.57	—	Survived
9	Non-diabetic	52	Adenovirus + RSV	5.88	3.03	2.75	—	Survived
10	Non-diabetic	61	Influenza A/H3 + SARS-CoV-2	5.09	2.93	0.48	—	Survived
11	Non-diabetic	88	RSV + SARS-CoV-2	3.90	28.68	7.08	Cardiovascular	Survived
12	Non-diabetic	28	Coronavirus HKU1 + Coronavirus NL63	3.26	2.67	4.31	Respiratory	Survived
13	Non-diabetic	75	Adenovirus + Influenza A	2.65	22.39	5.09	Respiratory	Survived
14	Non-diabetic	47	Parainfluenza + RSV	1.25	15.59	5.11	—	Survived
15	Diabetic	79	Rhinovirus + hMPV	266.20	446.64	3.51	Respiratory	Survived
16	Diabetic	82	Influenza A/H1N1-2009 + SARS-CoV-2	240.80	208.30	16.02	Renal	Died
17	Diabetic	85	Coronavirus OC43 + Influenza A/H1N1-2009	144.50	285.50	11.00	Renal	Survived
18	Diabetic	84	Coronavirus NL63 + Influenza A	36.44	80.83	3.29	—	Survived
19	Diabetic	77	Coronavirus OC43 + Influenza A	29.30	91.21	5.57	Respiratory	Survived
20	Diabetic	70	Rhinovirus + SARS-CoV-2	20.14	88.07	5.15	Respiratory	Survived
21	Diabetic	80	Influenza B + SARS-CoV-2	18.42	32.71	2.89	Cardiovascular, Renal	Survived
22	Diabetic	86	Influenza A + SARS-CoV-2	15.25	116.46	18.12	Cardiovascular	Survived

Abbreviations: CRP, C-reactive protein; hMPV, human metapneumovirus; IL-6, interleukin-6; NLR, neutrophil-to-lymphocyte ratio; RSV, respiratory syncytial virus. Non-diabetic and diabetic patients correspond to Groups 2 and 4, respectively. All viral pairs were identified by multiplex RT-PCR. Laboratory values are baseline (Day 1) measurements.

## 4. Discussion

In this prospective longitudinal cohort, a pre-existing diabetic background was associated with a disproportionately amplified inflammatory response to viral co-infection rather than a simply additive one. The higher IL-6 and CRP at Day 1 in co-infected patients with diabetes compared with co-infected patients without diabetes is compatible with an additional contribution of the diabetic metabolic and immunological background to the inflammatory response, beyond viral burden alone [15]. Vaccination coverage and sex distribution were similar across groups, but the co-infected subgroups were small (n = 8 and n = 14) and differed in age (median 81 vs. 56.5 years); the observed differences therefore cannot be attributed to diabetes with certainty and should be regarded as exploratory.

The mechanistic basis is multi-layered. Chronic hyperglycemia maintains circulating monocytes and macrophages in a hyper-reactive M1-polarized state through NLRP3 inflammasome activation and sustained NF-κB signaling [23]. Advanced glycation end-products bind RAGE receptors on innate immune cells, priming them for exaggerated cytokine release upon any subsequent stimulus. When two viral pathogens simultaneously trigger pattern recognition receptor pathways, TLR7/8 for RSV and TLR3 for SARS-CoV-2 [24,25,26], for example, the cumulative PRR activation in this pre-primed environment may exceed the negative feedback capacity of regulatory T cells and IL-10-mediated counter-signaling [8]. Our data are compatible with this saturation model: co-infection in non-diabetic hosts did not produce a higher median IL-6 than non-diabetic mono-infection (6.20 vs. 11.32 pg/mL, *p* = n.s.), which would be consistent with the diabetic background contributing to the amplified response [27,28,29,30]. However, the non-diabetic co-infected subgroup was small (n = 14) and younger than the other groups, and we cannot exclude that age, comorbidities, or chance contribute to this pattern.

Our mechanistic framing centered on chronic hyperglycemia and advanced glycation end-products, but the diabetic immunometabolic state is broader. Insulin resistance and dyslipidemia independently sustain low-grade inflammation and may contribute to the impaired inflammatory resolution we observed, beyond glucose alone. Cytokines with context-dependent, dual functions are also relevant: transforming growth factor-beta (TGF-β), which is activated by influenza neuraminidase, can be protective during influenza by limiting immunopathology, yet exerts both suppressive and pro-inflammatory effects on immune cells depending on the microenvironment, and shows cytoprotective anti-oxidative activity under hyperglycemia in some tissues. A shift in this balance in the diabetic host could plausibly influence whether the response to viral co-infection is resolving or deteriorative. These pathways were not measured here and represent a limitation and a direction for future immunophenotyping [31].

The Day 6 NLR dynamics are among the more notable findings of this study [15,32]. The Wilcoxon paired analysis showed that NLR in Group 4 failed to resolve between Day 1 and Day 6 (*p* = 0.2367), while NLR resolved significantly in Group 1 (*p* < 0.0001) and Group 3 (*p* = 0.0001), and the small Group 2 trended downward. This minimal NLR decline in the diabetic co-infected group is unlikely to be explained by Day 1 baseline differences (Group 4 NLR 5.36 vs. Group 2 3.81 was non-significant at admission, *p* = 0.0698). It is consistent with ongoing neutrophilic activation persisting beyond the acute viral replication phase. Such a pattern would be in keeping with cytokine-sustained neutrophil activity in a hyperglycemic environment, where the glucose-dependent oxidative burst and impaired neutrophil apoptosis create a self-perpetuating inflammatory loop. CRP showed a within-group decline in Group 4 (Wilcoxon *p* = 0.0234) and IL-6 trended in the same direction (*p* = 0.0547), which could be compatible with a preferentially cellular rather than humoral pattern of immune dysregulation; however, with eight patients and possible survivor selection at Day 6, this interpretation is tentative. Clinically, a persistently elevated Day 6 NLR in a diabetic co-infected patient may serve as a warning sign for secondary bacterial infection or ongoing tissue injury and warrants further study.

An emerging framework may help explain the delayed inflammatory resolution we observed. Chronic hyperglycemia can induce trained immunity, metabolic and epigenetic reprogramming of innate immune cells and their bone-marrow progenitors that produces a persistent hyper-inflammatory phenotype, sometimes termed hyperglycemic or metabolic memory, which persists even after glycemia is corrected. The exaggerated baseline IL-6 and the failure of NLR to resolve by Day 6 in our diabetic co-infected patients are compatible with such maladaptive trained immunity, in which a pre-programmed innate compartment over-responds to a viral challenge and resolves slowly. This remains a hypothesis in our cohort, as we did not perform epigenetic or functional immune profiling. It also implies that a single biomarker may be insufficient: combining IL-6 with complementary immunometabolic markers (e.g., procalcitonin, NLR, and glycemic indices such as HbA1c) into a composite index may capture this reprogrammed state better than IL-6 alone, and merits prospective evaluation [33,34].

The procalcitonin data are consistent with this interpretation. The 49-fold higher PCT in Group 4 at admission (1.95 vs. 0.04 ng/mL) in the context of a viral-confirmed infection, may reflect early secondary bacterial co-infection or mucosal barrier disruption. This is paralleled by the 2.5-fold higher antibiotic burden in Group 4 and links the inflammatory findings to antimicrobial stewardship in clinical practice. Current stewardship guidelines rely on PCT thresholds derived from general medical populations; our data suggest, in an exploratory sense, that PCT interpretation in diabetic patients with viral co-infections may need to account for higher baseline values, but this would need to be tested prospectively before any threshold adjustment could be recommended.

The stepwise respiratory failure escalation, 12.2% (Non-DM Mono) → 35.7% (Non-DM Co) → 24.6% (DM Mono) → 37.5% (DM Co), shows a notable pattern: non-diabetic co-infected patients (35.7%) had a higher rate of respiratory failure than diabetic mono-infected patients (24.6%). This suggests that the pulmonary parenchymal consequences of dual viral load can be substantial even in metabolically healthy hosts. In addition, the respiratory support escalation data, with diabetic patients requiring significantly more HFNO (7.9% vs. 1.8%, *p* = 0.0166), CPAP (6.2% vs. 0.9%, *p* = 0.0080), and orotracheal intubation (6.2% vs. 0.9%, *p* = 0.0207), independently corroborates the greater severity of immune-mediated lung parenchymal damage in the diabetic cohort beyond what organ-failure binary endpoints alone capture.

An exploratory output of this study is the identification of a candidate biomarker threshold. The ROC analysis identified baseline IL-6 > 55.78 pg/mL as the optimal cutoff for in-hospital mortality prediction (AUC 0.812, sensitivity 71.0%, specificity 79.1%). This threshold is approximately five-fold higher than the upper limit of normal in healthy controls and lies between the median values observed in non-diabetic (11.32 pg/mL) and diabetic mono-infected (44.80 pg/mL) patients in our cohort. The statistical superiority of IL-6 over both CRP (DeLong *p* = 0.0029) and NLR (DeLong *p* = 0.0001) is consistent: IL-6 is the principal upstream amplifier of the acute phase response, while CRP is its downstream consequence. The proposed threshold could be evaluated alongside risk-stratification tools such as the 4C Mortality Score and SOFA, providing a single-biomarker decision aid available at admission to support early intensification of monitoring or therapeutic escalation in high-risk patients.

Regarding the original hypothesis of strain-specific viral synergy, our cohort revealed an important epidemiological reality: with 22 co-infected patients distributed across 20 unique viral pairs, statistical testing of individual combinations is not feasible. This co-infection heterogeneity reflects the natural stochastic diversity of respiratory viral co-circulation in the post-pandemic era and is itself a novel epidemiological observation. Acknowledging that descriptive observations on eight patients cannot support statistical inference, the following patterns are reported as hypothesis-generating. SARS-CoV-2-containing viral pairs were responsible for three out of four multi-organ failure events (cardiovascular or renal) in the diabetic co-infected group, while non-SARS-CoV-2 pairs, including human Metapneumovirus + Rhinovirus and Influenza A/H1-2009 + Coronavirus OC43, drove the highest IL-6 peaks (266.20 pg/mL and 144.50 pg/mL, respectively). These case-level observations could be compatible with SARS-CoV-2-containing pairs preferentially involving endothelial and thrombo-inflammatory pathways, and non-SARS pairs producing more pronounced cytokine amplification, but the numbers are too small for this distinction to be drawn formally. These observations constitute a hypothesis-generating signal that warrants prospective investigation in larger multicenter cohorts.

Several limitations must be acknowledged. The DM Co-infected subgroup (Group 4, n = 8) is small, reflecting the low natural co-infection prevalence in this geographic cohort. While robust non-parametric tests protect against Type I errors from small samples, the statistical power for detecting subtle differences in this group is limited. Day 6 follow-up data was unavailable for 82 patients (19.1%), predominantly due to early discharge indicating clinical recovery; this non-random missingness may underestimate resolution rates. The ROC-derived IL-6 cutoff requires external validation in independent cohorts before clinical implementation. Finally, unmeasured confounders including specific DM complications, antidiabetic medication class effects, and oxygen therapy duration may partially explain observed associations.

Age warrants particular consideration, as it is a major determinant of both inflammatory response and mortality and, within the small co-infected subgroups, is inseparably linked to diabetes status (Group 4 median 81 years vs. Group 2 56.5 years); these subgroup comparisons therefore remain exploratory. However, in the full cohort the diabetes-associated risk was largely independent of age: after adjustment for age, sex, and body mass index, diabetes remained associated with an approximately seven-fold higher odds of in-hospital mortality (adjusted OR 6.98, 95% CI 3.15–15.48) and with substantially higher baseline IL-6 (+30.6 pg/mL) and CRP (+46.7 mg/L), and a sensitivity analysis confined to a common age band (65–80 years) reproduced these differences. Together, these findings indicate that the inflammatory and prognostic burden attributed to diabetes is not merely an age effect, while age remains an important independent contributor. Residual confounding by age or unmeasured comorbidities nevertheless cannot be fully excluded in the small co-infected subgroups.

## 5. Conclusions

In this single-center prospective cohort, hospitalized patients with diabetes had a more pronounced inflammatory profile and worse clinical course during viral respiratory infection than patients without diabetes, including in the small subgroup with viral–viral co-infection. Diabetes was associated with an amplified and more slowly resolving inflammatory response, reflected in 5.3-fold higher IL-6, 4.5-fold higher CRP, and minimal NLR resolution by Day 6. This profile was accompanied by a higher incidence of respiratory failure, greater need for HFNO/intubation requirements, acute renal strain (urea 82.39 vs. 28.89 mg/dL), and a 49-fold higher procalcitonin burden, collectively requiring a median of 2.50 concurrent antibiotic courses. In-hospital mortality reached 25.6% in diabetic versus 3.7% in non-diabetic patients, an approximately seven-fold mortality gradient (*p* < 0.0001).

ROC analysis identified baseline serum IL-6 > 55.78 pg/mL as a candidate threshold for in-hospital mortality prediction (AUC 0.812, sensitivity 71.0%, specificity 79.1%), outperforming CRP and NLR. Several clinical implications may follow. First, diabetic patients with confirmed viral co-infections may warrant closer monitoring and a lower escalation thresholds for anti-inflammatory therapy. Second, a Day 6 NLR failing to decline by ≥20% from Day 1 in co-infected diabetics may serve as an early warning sign for secondary bacterial infection. Third, baseline IL-6 above 55.78 pg/mL may help identify patients in whom earlier intensification of care should be considered. Validation in larger, multicenter cohorts with viral load quantification will be needed before these findings can be applied in practice.

On the basis of these exploratory findings, we would cautiously suggest that hospitalized diabetic patients with acute viral respiratory infection may benefit from early multiplex PCR characterization and a baseline IL-6 measurement at admission, to identify a higher risk subgroup for closer monitoring. This is a hypothesis for testing, not an established standard. Validation would require a prospective, multicenter study with a pre-specified, assay-standardized IL-6 threshold applied to an external cohort, age- and comorbidity-adjusted analysis, and assessment of whether early identification changes management and outcomes. Cost-effectiveness of routine multiplex testing in this population would also need to be established before routine adoption.

## Figures and Tables

**Table 1:** Baseline demographic and clinical characteristics stratified across the four host–pathogen cohorts.

Patient Characteristics	Group 1 Non-DM Mono (n = 205)	Group 2 Non-DM Co-Inf (n = 14)	Group 3 DM Mono (n = 203)	Group 4 DM Co-Inf (n = 8)	Global *p*-Value
Age, years, median (IQR)	66.0 (45.0–77.0)	56.5 (37.0–75.0)	72.0 (66.0–78.0)	81.0 (78.0–84.5)	<0.0001
BMI, kg/m^2^, median (IQR)	25.1 (22.5–29.3)	26.0 (22.1–27.7)	29.4 (24.9–34.6)	27.6 (25.7–29.4)	<0.0001
Abdominal circumference (cm)	89.0 (73.8–100.0)	84.0 (75.0–98.0)	100.0 (88.0–114.0)	97.0 (88.5–105.0)	<0.0001
HbA1c, %, median (IQR)	5.2 (5.1–5.4)	5.3 (5.1–5.5)	7.2 (6.5–9.1)	9.2 (7.8–10.5)	<0.0001
Admission glycemia, mmol/L	5.6 (4.9–6.8)	5.4 (4.8–6.1)	9.6 (6.7–13.4)	10.8 (7.8–14.2)	<0.0001
Length of stay, days	6.0 (5.0–8.0)	6.0 (3.0–7.0)	9.0 (6.0–12.8)	8.0 (7.0–11.0)	<0.0001
Male sex, n (%)	91 (44.4%)	6 (42.9%)	102 (50.2%)	5 (62.5%)	0.5219
COVID-19 vaccinated, n (%)	103 (53.6%)	6 (60.0%)	109 (53.7%)	7 (87.5%)	0.2904
Influenza vaccinated, n (%)	38 (19.9%)	2 (20.0%)	35 (17.3%)	3 (37.5%)	0.5213

IQR: interquartile range; BMI: body mass index; HbA1c: glycated hemoglobin; DM: diabetes mellitus. Continuous variables compared by Kruskal–Wallis H test; categorical variables by chi-square test.

**Table 2:** Host immune biomarkers and inflammatory kinetics across baseline (Day 1) and resolution checkpoints (Day 6), stratified by group.

Biomarker (Median, IQR)	Group 1 Non-DM Mono	Group 2 Non-DM Co	Group 3 DM Mono	Group 4 DM Co	KW *p*-Value	MW Pairwise (Gr.4 vs. 2)
Baseline IL-6, pg/mL	11.32 (4.38–26.31)	6.20 (3.90–14.55)	44.80 (14.73–88.68)	32.87 (19.28–192.65)	<0.0001	0.0006
Day 6 IL-6, pg/mL	6.65 (2.23–12.10)	6.13 (1.44–9.29)	21.96 (8.90–61.12)	12.01 (10.51–47.72)	<0.0001	0.0183
Baseline CRP, mg/L	38.24 (8.70–105.40)	23.03 (7.32–50.51)	82.33 (31.32–171.58)	103.83 (84.45–246.90)	<0.0001	0.0012
Day 6 CRP, mg/L	11.15 (5.17–23.29)	11.60 (5.29–27.91)	23.27 (8.38–57.93)	10.79 (9.02–117.79)	<0.0001	0.6058
Baseline NLR	4.47 (2.13–9.36)	3.81 (2.57–4.90)	6.83 (3.39–12.62)	5.36 (3.40–13.51)	<0.0001	0.0698
Day 6 NLR	3.24 (1.94–7.25)	2.57 (1.12–4.83)	4.19 (2.65–10.48)	5.24 (3.07–7.69)	0.0054	0.2766

KW: Kruskal–Wallis test (global, 4 groups); MW: Mann–Whitney U test (Group 4 vs. Group 2 pairwise, exact method, two-sided). NLR: neutrophil-to-lymphocyte ratio.

**Table 3:** Multi-systemic outcomes, mortality, respiratory support requirements, antibiotic burden and procalcitonin/renal parameters across the four cohorts.

Clinical Parameter	Group 1 Non-DM Mono	Group 2 Non-DM Co	Group 3 DM Mono	Group 4 DM Co	Global *p*-Value	MW Pairwise (Gr.4 vs. 2)
Procalcitonin Day 1, ng/mL	0.08 (0.04–0.27)	0.04 (0.03–0.07)	0.48 (0.12–2.11)	1.95 (0.21–7.41)	<0.0001	0.0029
Urea Day 1, mg/dL	36.38 (27.29–57.78)	28.89 (23.54–34.24)	68.48 (43.34–93.63)	82.39 (54.57–110.21)	<0.0001	0.0026
Antibiotic count, n	1.0 (0.0–2.0)	1.0 (0.0–2.0)	2.0 (1.0–2.0)	2.5 (2.0–4.0)	<0.0001	0.0036
Respiratory failure, n (%)	25 (12.2%)	5 (35.7%)	50 (24.6%)	3 (37.5%)	0.0023	—
Cardiovascular failure, n (%)	5 (2.4%)	1 (7.1%)	37 (18.2%)	2 (25.0%)	<0.0001	—
Renal/urinary failure, n (%)	27 (13.2%)	0 (0.0%)	59 (29.1%)	3 (37.5%)	0.0001	—
In-hospital mortality, n (%)	7 (3.4%)	1 (7.1%)	53 (26.1%)	1 (12.5%)	<0.0001	—
CPAP, n (%)	2 (1.0%)	0 (0.0%)	13 (6.4%)	0 (0.0%)	0.0080	—
HFNO, n (%)	3 (1.5%)	1 (7.1%)	16 (7.9%)	1 (12.5%)	0.0166	—
Orotracheal intubation, n (%)	2 (1.0%)	0 (0.0%)	13 (6.4%)	0 (0.0%)	0.0207	—

Continuous variables (median, IQR) compared by Kruskal–Wallis (global) and Mann–Whitney U (pairwise G4 vs. G2, exact). Categorical variables compared by chi-square test (Fisher’s exact applied when expected cell counts < 5). DM: diabetes mellitus; PCT: procalcitonin; HFNO: high-flow nasal oxygen; MV: mechanical ventilation.

**Table 4:** ROC curve analysis for prediction of in-hospital mortality using baseline inflammatory biomarkers (n = 430; 62 deaths, 14.4%).

Biomarker	AUC	95% CI	Optimal Cutoff (Youden Index)	Sensitivity/Specificity	*p*-Value
IL-6 Day 1	0.812	0.772–0.848	>55.78 pg/mL	71.0%/79.1%	<0.0001
CRP Day 1	0.706	0.660–0.748	>81.1 mg/L	77.4%/64.4%	<0.0001
NLR Day 1	0.656	0.609–0.701	>4.22	80.7%/43.5%	<0.0001

AUC: area under the receiver operating characteristic curve; CI: confidence interval (DeLong method); optimal cutoff identified by Youden index J. DeLong pairwise comparisons: IL-6 vs. CRP *p* = 0.0029; IL-6 vs. NLR *p* = 0.0001; CRP vs. NLR *p* = 0.1920.

## Data Availability

Data availability is subject to hospital approval (due to internal regulations of the hospital—Regulation UE nr. 679 from 2016 regarding protection of personal data).

## Abbreviations

The following abbreviations are used in this manuscript:

AGEs	Advanced Glycation End-products
AUC	Area Under the ROC Curve
CPAP	Continuous Positive Airway Pressure
CRP	C-Reactive Protein
DM	Diabetes Mellitus
HFNO	High-Flow Nasal Oxygen
IL-6	Interleukin-6
IQR	Interquartile Range
NLR	Neutrophil-to-Lymphocyte Ratio
PCT	Procalcitonin
PCR	Polymerase Chain Reaction
PRR	Pattern Recognition Receptor
ROC	Receiver Operating Characteristic
RSV	Respiratory Syncytial Virus
SARS-CoV-2	Severe Acute Respiratory Syndrome Coronavirus 2
